# Rapid sequestration of rock avalanche deposits within glaciers

**DOI:** 10.1038/ncomms8964

**Published:** 2015-08-19

**Authors:** Stuart A. Dunning, Nicholas J. Rosser, Samuel T. McColl, Natalya V. Reznichenko

**Affiliations:** 1 Department of Geography, Northumbria University, Newcastle upon Tyne NE1 8ST, UK; 2 Geography Department and IHRR, Durham University, Durham DH1 3LE, UK; 3 Massey University, Palmerston North, New Zealand

## Abstract

Topographic development in mountainous landscapes is a complex interplay between tectonics, climate and denudation. Glaciers erode valleys to generate headwall relief, and hillslope processes control the height and retreat of the peaks. The magnitude–frequency of these landslides and their long-term ability to lower mountains above glaciers is poorly understood; however, small, frequent rockfalls are currently thought to dominate. The preservation of rarer, larger, landslide deposits is exceptionally short-lived, as they are rapidly reworked. The 2013 Mount Haast rock avalanche that failed from the slopes of Aoraki/Mount Cook, New Zealand, onto the glacier accumulation zone below was invisible to conventional remote sensing after just 3 months. Here we use sub-surface data to reveal the now-buried landslide deposit, and suggest that large landslides are the primary hillslope erosion mechanism here. These data show how past large landslides can be identified in accumulation zones, providing an untapped archive of erosive events in mountainous landscapes.

## Abstract (web summary)

Small, frequent rockfalls are thought to dominate the erosion of mountains above rockfalls, and little is known about rare large landslides as material is rapidly reworked. Here, the authors present sub-surface data from a large rock avalanche showing how such landslides can be recognized from their deposits.

## Graphical abstract

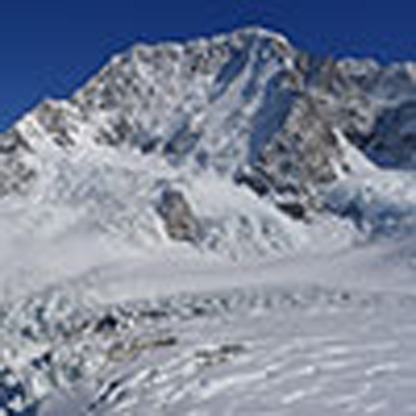

Mountainous relief is generated by the interaction of tectonics and climate^1^, with the balance responsible for the net change of a landscape's relief and elevation. The potential of rivers and glaciers to erode (or protect) mountainous landscapes in response to tectonic and climatic forcing has been the focus of much work^1^,^2^, with hillslope processes often assumed to respond to, and reflect, undercutting by either process. These hillslope processes undertake the geomorphic work above rivers and ice that lowers mountain peaks and retreats valley sides^3^, providing sediment to be mobilized from orogens^4^, therefore playing a key role in controlling relief and elevation. The ability of landslides to transfer sufficient mass to keep pace with fluvial and glacial downcutting and tectonic uplift is not well constrained and is dependent upon characterizing their long-term magnitude–frequency^5^,^6^.

Globally, the largest landslides are thought to be responsible for 1–10% of Late Pleistocene to Holocene erosion (≥1 mm kyr^−1^) in active mountain belts^3^. These events are clustered in the steepest terrain, which often corresponds to the walls of glaciated valleys. However, our information is incomplete: data are heavily censured by fluvial^4^ and glacial reworking^5^,^7^,^8^; failures can be buried by snowfall and ingested into crevasses before emergence in the ablation zone^6^, or they can be transported entirely on the surface to deposition^9^,^10^,^11^,^12^. We are often reliant on preserved deposits in ice-free areas, and our records are best in more arid, high-mountain regions^7^,^8^.

There is evidence of re-adjustment of glacially sculpted landscapes by landsliding as ice retreats^13^,^14^,^15^,^16^, which is termed paraglacial. There is also an apparent close contemporary association of warmer temperatures and increased mass wasting from glacial peaks^17^,^18^, although large landslides seem to lag^19^. We rely heavily on the assumption of a direct link between erosion and climate: the sediment sourced from above the contemporary glaciers contributes to oceanic sedimentation, which is often used to link climate to erosion^20^, and the moraines generated in periods of enhanced mass wasting are used as climatic indicators^11^. Understanding the interaction between large landslides onto ice and glacial dynamics is crucial for determining the palaeoclimatic significance of landslide-sediment-dominated moraines^5^,^11^,^21^,^22^. Over these longer time scales, slopes above glacier ice are thought to respond to a ‘glacial buzzsaw' that operates to limit peak heights, with the result being a correspondence of mean topographic elevations with Cenozoic equilibrium line altitudes^2^,^23^,^24^,^25^.

Within landscapes dominated by contemporary glacial erosion, we lack reliable data on the timing and rates of landslide activity to enable extrapolation to such time scales, especially in glacier accumulation areas, which, by area, dominate glacial terrain^9^. Glacial erosion rapidly denudes valley floors, steepening valley sides above^23^,^26^,^27^, which at some point must fail. Modern estimates of the sediment flux from hillslope processes in these landscapes vary widely^28^,^29^, from 24 to 60%. The relative importance of near-continuous mass wasting by small rockfalls as compared with the less-frequent large-scale mountain-face collapses to these rates is unknown. Much emphasis has been placed on change driven by headwall retreat through small but frequent periglacial rockfall^25^,^26^,^30^,^31^ and short-term data sets^32^ achieve erosion of 0.002–5 mm per year. However, short-term rates are often judged insufficient to accumulate to long-term denudation^31^,^32^. Large low-frequency landslides are a major component of this difference; yet, as they are often rapidly sequestered in accumulation zones, they have been hard to quantify^31^,^32^.

In the deglaciating Chugach mountains of Alaska, paraglacial erosion rates from large landslides alone of 0.7–7 mm per year at a single glacier^33^ and 0.5–0.7 mm per year over a 50 per year period for a much wider area^6^ have been estimated. In the deglaciated region of Western Greenland (Disko Island), cirque rockwall retreat rates of 3–5 mm per year from periglacial rockfalls have been estimated^34^; yet, on a 30-km stretch of the nearby Nuussuaq coastline, a cluster of rock avalanche deposits^35^ have been shown to have eroded at rates of around 3 mm per year. In the Mt Blanc massif, at least 19 large landslides have occurred onto ice since 2500 BP (before present, which for radiocarbon dating equates to number of years before 1950), and they are thought to be becoming more common as climate warms and permafrost thaws^17^. In some locations of concentrated study, larger, rarer landslides therefore appear to be able to erode at long-term rates similar to the frequent, small, periglacial rockfalls.

Wider study to quantify their contribution to limiting relief above ice has been limited by our ability to identify the deposits of large landslides in glacial terrain, especially when they enter into the accumulation areas of glaciers. It is likely that many large landslides have not been resolved within both our contemporary and palaeo records. Our records of landslides in high mountains are ‘…too short, too local, or are [too] biased'^35^,^36^ to assess the rates and timing of landslides, as well as any changes as ice retreats and thins and permafrost recedes in a warming climate^18^. The reworking of deposits presents a challenge for documenting the significance of landslides, particularly given the low recurrence of suitable conditions for repeat satellite imaging^6^. Source scars are often misidentified as cirques^37^ or not recognized as discreet landforms at all^8^,^38^,^39^; reliably finding the dispersing landslide deposits is key.

In this work, we start to address this challenge using data collected at the site of the 2013 Mt Haast rock avalanche deposit, New Zealand, which, 1 year after emplacement, is fully sequestered into the glacier it deposited onto. We use satellite imagery to highlight the rapid loss of supraglacial deposits to conventional remote sensing, and a ground-penetrating radar (GPR) survey to find the dispersing deposit. Using GPR, the buried deposit is clearly resolved under fresh snow accumulation, as are several smaller, previously unknown rockfall deposits. We use these data to discuss the wider relevance of these interpretations for the erosion of mountains by large landslides.

Landslides are commonly observed in the Sothern Alps of New Zealand^15^,^16^,^40^,^41^,^42^, and rock avalanches seem, at least anecdotally, to be occurring more frequently^42^ from a late Holocene average of 1 in 100 years, a 1976–1999 average of 4 in 10 years, to 20 in the past decade. In total, ∼14.7 × 10^6^ m^3^ of bedrock has been removed from the slopes of Aoraki/Mt Cook, New Zealand's highest peak (3,724 m above sea level (m.a.s.l.)), and its surrounding ridges (Fig. 1) by rock avalanches over the period of 23 years. Three have been observed, or found soon after failure, in 1991, 2013 and 2014. The 1991 11.8 × 10^6^ m^3^ rock avalanche lowered the summit of Aoraki by ∼30 m, depositing debris onto the Grand Plateau Glacier to the east, before travelling down the Hochstetter Icefall onto the Tasman Glacier below^40^. On 21st January 2013, a rock avalanche failed from the ridge between Mt Haast and Mt Dixon (3,040 m.a.s.l.). Around 2 × 10^6^ m^3^ of rock, snow and ice travelled 2.9 km from the source, running out over the northern margins of the Grand Plateau, stalling close to the top of the Hochstetter Icefall (Fig. 2). Several eye witnesses captured the event on film^43^ from Plateau Hut, allowing flow velocity estimates to be made (160 km h^−1^) (ref. 44). The final deposit was estimated to be 6–7 m thick and displayed classic rock avalanche features including lobes and levees comprising a mix of poorly sorted debris ranging up to large boulder size (>10 m^3^) (ref. 44). In July 2014, a 0.9 × 10^6^-m^3^ rock avalanche sourced from the Hillary Ridge (south ridge) of Aoraki ran out to the west, down onto the Hooker Glacier surface, damaging Gardiner Hut^42^.

## Results

### Time-series remote sensing

**Figure 1:**
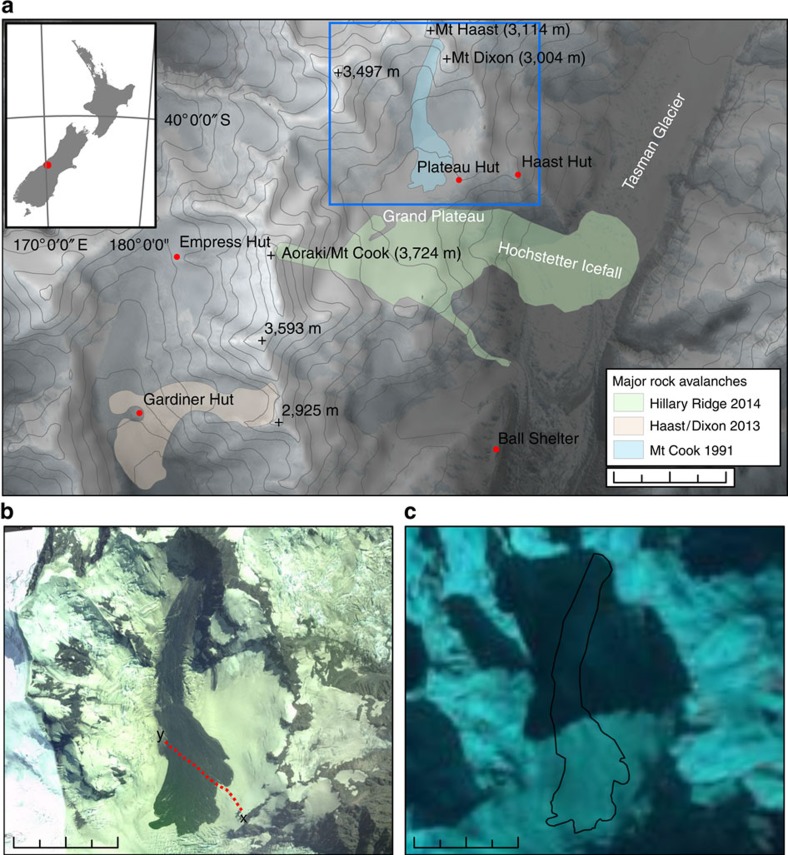
Recent rock avalanches from the ridges of Aoraki/Mt Cook. (**a**) Hillshaded elevation model of the Mt Cook area showing recent rock avalanche deposit outlines as emplaced. (**b**) NASA EO-I ALI Satellite image of the landslide deposit captured on 02/13/13, with GPR profile marked. (**c**) NASA Landsat 7 image captured on 5 November 2013, in which no evidence of the landslide is visible, *in situ* deposit outline for context. Scale bars in (**a**) is 2 km, and in (**b**) and (**c**) 1 km.

The initial Mt Haast deposit is visible in post-event satellite imagery for only 3 months. Onwards from the 11 May 2013 image, snow cover fully obscures the deposit, and no chromatic contrast is apparent on the Grand Plateau surface to indicate a surface texture different from that of the surrounding glacier (Fig. 1). Although an effect moderated by season, and cloud cover in satellite imaging, the removal of the surface expression of the landslide is remarkably rapid considering the initial rock avalanche deposit volume and extent.

In an image from 17 April 2013, sediment is visible in the crevasse field at the crest of the Hochstetter Icefall, corresponding to the leading edge of the now englacial rock avalanche deposit. The icefall has flow rates of up to ∼300 m per year and an overall length of 2 km (ref. 45); as it transits into and through the icefall, the 2013 debris is being rapidly reworked, mixed and disaggregated from its original sheet-like form through extensional flow, extensive crevassing and frequent serac collapse. Field photography of the icefall in April 2014 (Fig. 3a) revealed much debris at depth within crevasses, illustrating the potential, in extensional zones, for vertical mixing and incorporation of englacial debris through a glacier.

### GPR data—100- and 50-MHz survey

In April 2014, at the time of the survey reported here, the deposit was entirely buried beneath the snow/firn cover, leaving no topographic expression of the deposit at the snow surface. The buried deposit was visible in crevasses, in the Hochstetter Icefall, in the Grand Plateau and in the icefall beneath Mt Haast, at depths estimated to be in the order of 5–10 m (Fig. 3). This tallies with an estimated 15-month snow/firn accumulation of 4–5 m from 7,000 mm average annual precipitation, measured^46^,^47^ at the nearby upper Tasman Glacier (2,200 m.a.s.l.).

We interpret a sharply dipping interface between the overlying snow cover (at the edge) and ice in the south-eastern end of the radar transect as bedrock in the 50-MHz data (Fig. 4). This interface is either obscured by noise or out of range because of time-window limits beyond 70 m along the radar transect. The bedrock interface does not re-appear at the south-western end of the transect. Both the 50- and 100-MHz data show what we interpret as the rock avalanche deposit beneath the 2013/14 snow accumulation (Fig. 4), which tallies directly to the observations of deposit depth seen in crevasses (Fig. 3). The upper surface of the landslide deposit is characterized by a reflector with abundant hyperbolas, consistent with the field data of the deposit surface comprising highly angular boulders of fragmented bedrock^44^. The 100-MHz profile images the deposit beneath this coarse boulder layer, and an indistinct lower boundary. We use this for thickness measurements, but we caution against defining this as an absolute landslide base because of signal attenuation and scattering through the debris, and the likely similarity in dielectric properties of a snow and an ice-rich basal shear as often found at the base of supraglacial landslides^5^,^10^,^40^,^48^. The deposit thickness (1–2 m) appears broadly consistent along the length of the transect, with a thicker (5–7 m) ∼170-m-wide region at the south-east end of the profile. The fresh landslide deposit in this area was estimated to comprise equal proportions of debris and ice entrained during runout, with a thickness measured at 0.5–7 m, and becoming thicker and more ice-rich towards the distal margin^44^.

**Figure 2:**
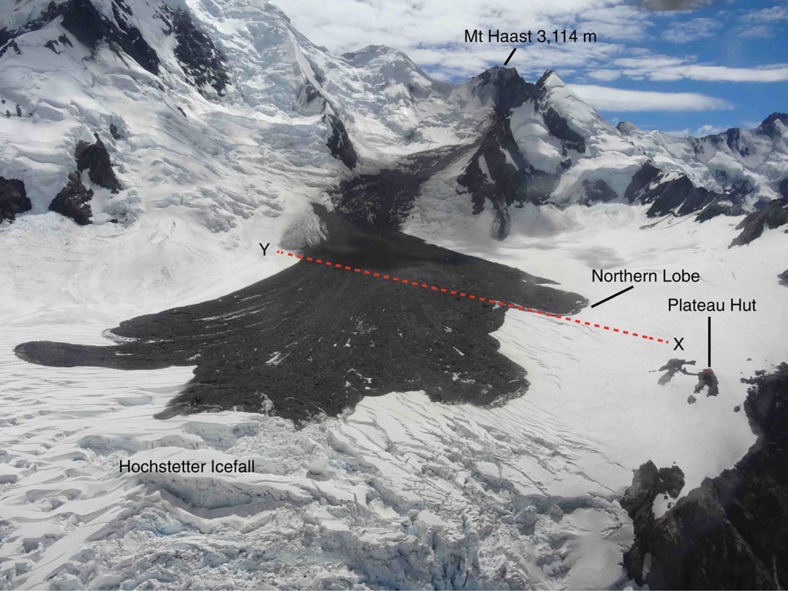
The 2013 Mount Haast rock avalanche deposit. The image was taken 25 days after failure and shows features discussed in the text. X–Y is the approximate position of the 50-MHz radar line (Fig. 4). Source: Charlie Hobbs, Southern Alps Guiding, New Zealand.

Our data are consistent with original field observations, but they also illustrate that the basal area and interior of the deposit lack the abundant GPR hyperbolas seen on the upper surface; we take this as being indicative of less-coarse debris in the basal zone where snow/ice entrainment would have been concentrated. We interpret the thicker area at the north-east end of the line as the Northern Lobe—an area that was observed^44^ to be thicker, and visibly ice and boulder rich. The upward-turned lateral margin is consistent with bulldozing/ploughing of the leading edge of the rock avalanche as it travelled, and the formation of a distal rim, observed at similar supraglacial rock avalanche deposits^10^, reported after^44^, and filmed during this event^43^. The location in these data of the margin as compared with the *in situ* deposit allows calculation of 30–70 m of horizontal movement since emplacement (Fig. 1), the error being associated with the original satellite image resolution and spatial referencing. At 480–550 m along the section, increased penetration is achieved, and hyperbolas are sparser; this is consistent with a less boulder-rich zone, or an area of increased snow and ice entrainment. It was reported^44^ that parts of the deposit were thin enough, and/or lacking in rock debris, such that the underlying glacier surface was visible. The inferred deposit thins out at the north-western end of the profile (Fig. 4), at a location consistent with moving off the *in situ* main deposit and onto a thin minor lobe, which on photographs (Fig. 2) appeared to be a thin dust layer (commonly observed to settle after the main landslide ceases^40^,^42^) with much visible snow.

The overlying 2013/14 snow and firn are clearly delimited as a multilayered drape over the rock avalanche deposit, filling topographic hollows in the deposit surface with up to 10 m of snow (Fig. 4). However, in places the deposit was established from the GPR data to be within 3–4 m of the surface, highlighting the influence of windblow and the local and original deposit topography on burial rates.

There are several isolated strong hyperbolas underneath the 2013 deposit, with several clusters at ∼20–30 m depth towards the S–W end of the transects (Fig. 4). The shapes of the hyperbolas and collapse of the hyperbolas (by migrating the data) indicate that they are narrow point sources, indicative of small objects, rather than large conduits or crevasses that would produce a broader hyperbolic shape. They are likely to be rock debris, and thus, although there is no evidence for other large events in our survey line, there is evidence to suggest one or more, older, undocumented, small rockfall deposits.

## Discussion

Determining the impact of climate change on slopes above retreating and thinning glacier ice, and their contribution to the ‘glacial buzzsaw', is reliant on characterizing hillslope processes over sufficiently long time scales to capture major contributing events. Despite the accumulation areas of glaciers dominating these landscapes by area^9^, these potential archives of large landslide-derived hillslope flux have not been investigated. We have shown here, using the January 2013 rock avalanche onto the Grand Plateau beneath Aoraki/Mt Cook, that GPR can be used to measure the deposit burial depth and thickness, to characterize the morphology indicative of a rock avalanche and to document the distribution of englacial landslide deposits. The 2013 rock avalanche deposit is the only significant reflector identified in the upper ∼50 m of ice surveyed, suggesting that it is the only major input of supraglacial sediment in the last 5–20 years to this portion of the Grand Plateau. This is consistent with historical records of major landslides in this area (Fig. 1). A number of smaller rockfall deposits, several hundred metres from the headwalls, at depths consistent with emplacement in the last 10–20 years suggest that even small debris inputs can easily be detected.

**Figure 3:**
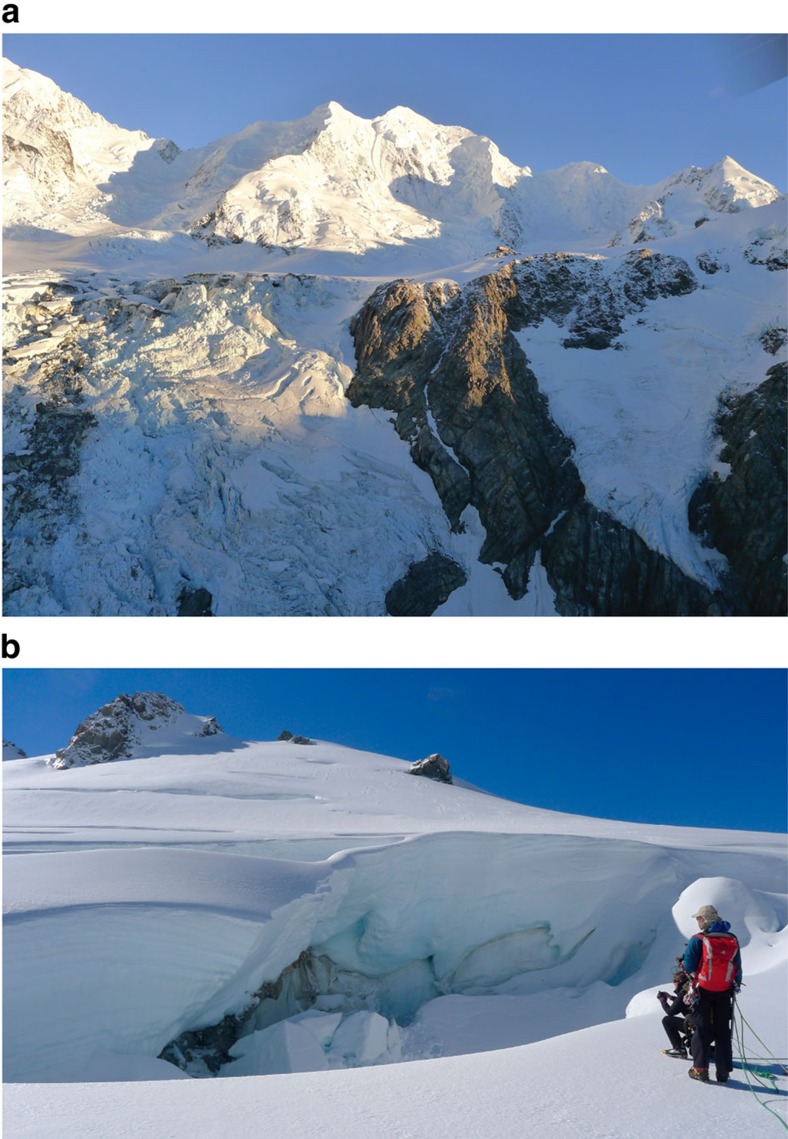
Reworked rock avalanche debris emergence. (**a**) View from the helicopter above Tasman Glacier looking north west at rock avalanche debris in the Hochstetter Icefall, with none visible on the Grand Plateau behind; Plateau Hut to the right for scale. (**b**) View from Grant Plateau looking north east (up flow) at rock avalanche debris in a crevasse on the inferred Eastern deposit margin buried beneath the 2013/14 snow and firn. The debris thins to a dust layer to the right.

**Figure 4:**
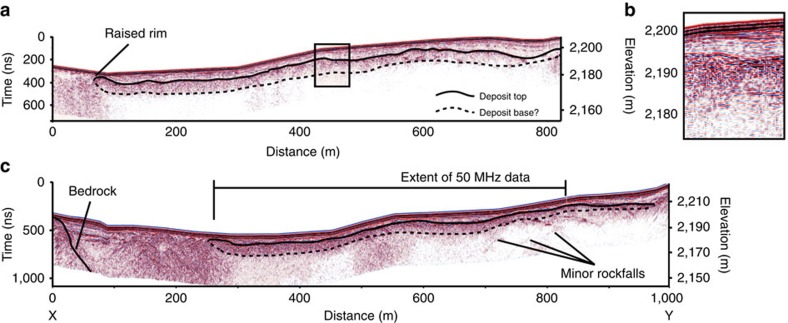
GPR data obtained from the Grand Plateau. (**a**) The 100-MHz data with snow layers overlying the 2013 rock avalanche deposit showing raised lateral rim, variable thickness and a poorly resolved base due to snow/entrainment. (**b**) Zoomed portion of (**a**) highlighting the dielectric contrasts between snow layering in the upper third and the clear rock avalanche surface reflector at 2,193 m. (**c**) The 50-MHz data showing the longer profile as marked in Fig. 1 with penetration to bedrock at the south-eastern end of the survey. Hyperbolas at depth relate to non-rock avalanche debris point returns and are interpreted as minor, older rockfalls.

The relatively low gradient and ice-flow velocities, and the resulting short annual transport distances in the central Grand Plateau area have rafted the deposit relatively intact within the glacier. Although our observations may appear simple and are limited to 14 months since debris emplacement, to date, the potential to find englacial rock avalanche deposits in accumulation zones has not been demonstrated. Because of the strong reflection produced by relatively thick debris, and the diagnostic features indicative of rock avalanche deposits (coarse clastic carapace, lobate pressure ridges and distal rims^10^), it is likely that parts of the deposit will remain visible to radar imaging after many years of additional snow accumulation. Major disruption of the deposit morphology is only seen as it enters the crevasses of the Hochstetter Icefall (Fig. 3).

Current methods to document the role of landslides in glacial landscapes rely on the identification of landslide deposits before entrainment, and currently there is no way of tracking the englacial transport of landslide debris before it subsequently emerges in the ablation zone as supraglacial debris^9^,^49^,^50^. Our approach fills this data gap, and it can track englacial debris transport of large landslides through accumulation zones; such an approach has previously only been conceptualized for the purposes of distinguishing between sources of englacial debris^51^. Using GPR on large deep accumulation zones gives the potential to look down into stacked archives of englacial rock avalanches. This is important, as the accumulation zone of some glaciers can account for as much as 95% by area (Hubbard Glacier, Alaska, for example), with the normal range when a glacier is in equilibrium somewhere between 40 and 80% (ref. 13). Mapping such deposits will aid our knowledge of when, how frequently and from where such events occur.

The frequency of rock avalanches has seemingly increased in recent times in our study region^42^. It is not clear whether this simply represents poor observations and deposit preservation over increasing time scales rather than some signal of climate change/paraglacial adjustment^14^,^15^,^16^,^19^, controlled by, in particular, permafrost degradation^18^, or increasing rock damage caused by tectonic strain accumulation towards the end of the Alpine Fault cycle^52^. Over geological time, the Mt Cook area represents a notable departure from being an effective glacial buzzsaw^27^, unable to keep pace with rapid uplift, whereby hillslopes are long and steep, possibly at threshold stability conditions^24^, and with relief slightly higher than expected^27^. Given the volumes of rock avalanches in the area, the scarcity of non-rock avalanche debris in the GPR data presented, the lack of reporting of frequent (daily) rockfall from the steep slopes observed in comparable locations^17^ and the protective covering^24^ of ice on the slopes, we advance the view that rock avalanches are the primary agents of hillslope erosion here.

On the basis of a successful demonstration of GPR for identifying features indicative and characteristic of rock avalanches, the application of sequential GPR (especially airborne-mounted GPR capable of surveying greater areas) provides an opportunity to develop a time series of englacial landslide debris dispersion through glacial systems. This approach has the potential to track the dispersion of buried landslides, and in doing so to use the deposit as an easily identifiable four-dimensional flow tracer/isochrone within the glacier in the period before exhumation farther down-glacier^9^. We do not yet know how a large landslide deposit moves through a glacier. Smaller rockfall events have been interpreted elsewhere to be transported in coherent lenses of material, before becoming folded and sheared, and later contributing significantly towards medial moraine formation^49^. Others have linked small-scale rockfalls ingested as crevasse fill in the accumulation zone to more widespread debris cover in the ablation zone of thinning glaciers^50^. Continuous debris sheets sourced from more spatially limited rockfall additions have been shown to transit through two cold-based Antarctic Dry Valley glaciers as coherent bands^51^. The emergence of large volumes of supraglacial debris has been linked to past accumulation zone rock avalanches in several mountain ranges^6^,^9^,^53^.

Current modelling^21^,^22^ has shown that ablation zone landslide deposits can effectively shut off ablation and alter glacial dynamics^11^,^12^,^48^. Such modelling has not been carried out for englacial rock avalanches. Rock avalanche debris has densities usually approaching twice that of the surrounding glacier ice, and given that their supraglacial volumes after emplacement are of a minimum of 1 × 10^6^ m^3^, such deposits may, in some instances, influence glacier flow and composition when travelling englacially.

Even large-volume, spatially extensive rock avalanches are rapidly subsumed within glaciers, notably within accumulation zones, leaving no visible surface expression after periods of days to months. With attempts to monitor the occurrence of remote landslides automatically still in development^54^, our understanding of the frequency and hazard (given how close all three Aoraki/Mt Cook landslides have come to climbing huts for example) posed by such events is limited. We show the good preservation of a rock avalanche deposit as it becomes encased within the ice of a glacier accumulation zone within 1 year of emplacement. As accumulation zones can be large proportions of a glacier's volume and area, they therefore hold valuable archives of hillslope process history that may be linked to the relief of mountainous terrain, and changing climatic drivers (and glacier health) if rates of large landslides truly increase as climate warms.

## Methods

### Rock avalanche deposit identification and tracking

We use an existing map^44^ based on an image captured on 5 February 2013 by the Advanced Land Imager (ALI) on NASA's Earth Observing-1 (EO-1), and the author's field and oblique aerial photo maps for the *in situ* deposit location and properties. Time-series satellite remote sensing used Landsat (USGS/NASA) imagery of nominal 30-m (false colour) and 15-m (panchromatic) resolution where the deposit area was free of cloud. To resolve post-depositional change and time to burial, we use sequential Landsat 7 and 8 imagery of 30-m resolution from the time of failure onwards. We used these data to select the locations for geophysical investigation using GPR, as shown in Fig. 2. The survey crossed in front of the *in situ* position of what was termed the Northern Lobe^42^, and then continued across the full width of the deposit.

### Ground-penetrating radar

An extensive review of the theoretical background to GPR and its application to snow and ice is provided elsewhere^55^. Here we emphasize that this technique has been extensively used on snow and ice and ice-debris mixes, including on rock glaciers. Ice thickening driven by supraglacial rock avalanche deposits in ablation zones has also been demonstrated^48^ using GPR, which penetrated through the debris, because of the dielectric contrast between landslide debris and snow/ice. To characterize the buried rock avalanche surveyed, we rely on the dielectric contrast between rock avalanche debris, the overlying snow and the glacier ice beneath. A common-offset survey technique was used with antennas of 100 and 50 MHz, deployed at 1- and 2-m separations, respectively. GPR profiles underwent time-zero correction, dewow, ACG gain adjustment and topographic correction using post-processed differential-GPS elevation data. Observed depths to the landslide deposit in crevasses and hyperbola fitting allowed for depth/time corrections to be calculated. The velocity obtained was ∼0.15 m ns^−1^, and this value was adopted for correcting the depth axis scale in the radargrams. All GPR transects were subject to crevasse-induced noise, with the dominant trend of crevassing running parallel to the survey line, and strong attenuation underneath the avalanche debris making structures beneath the avalanche debris difficult to identify. What we present is probably an underrepresentation of deeper features (below the landslide deposit), and a bedrock reflector being absent for large portions is not a definitive measure of minimum ice depths for this location.

## Additional information

**How to cite this article**: Dunning, S. A. *et al.* Rapid sequestration of rock avalanche deposits within glaciers. *Nat. Commun.* 6:7964 doi: 10.1038/ncomms8964 (2015).
